# NKp46 Clusters at the Immune Synapse and Regulates NK Cell Polarization

**DOI:** 10.3389/fimmu.2015.00495

**Published:** 2015-09-25

**Authors:** Uzi Hadad, Timothy J. Thauland, Olivia M. Martinez, Manish J. Butte, Angel Porgador, Sheri M. Krams

**Affiliations:** ^1^Department of Surgery, Division of Abdominal Transplantation, Stanford University, Stanford, CA, USA; ^2^The Shraga Segal Department of Microbiology and Immunology and Genetics, Ben-Gurion University of the Negev, Beersheba, Israel; ^3^Department of Pediatrics, Division of Immunology, Stanford University, Stanford, CA, USA

**Keywords:** cellular activation, cytoskeleton rearrangement, cytotoxicity, NKp46, immune synapse

## Abstract

Natural killer (NK) cells play an important role in first-line defense against tumor and virus-infected cells. The activity of NK cells is tightly regulated by a repertoire of cell surface expressed inhibitory and activating receptors. NKp46 is a major NK cell-activating receptor that is involved in the elimination of target cells. NK cells form different types of synapses that result in distinct functional outcomes: cytotoxic, inhibitory, and regulatory. Recent studies revealed that complex integration of NK receptor signaling controls cytoskeletal rearrangement and other immune synapse-related events. However, the distinct nature by which NKp46 participates in NK immunological synapse formation and function remains unknown. In this study, we determined that NKp46 forms microclusters structures at the immune synapse between NK cells and target cells. Over-expression of human NKp46 is correlated with increased accumulation of F-actin mesh at the immune synapse. Concordantly, knock-down of NKp46 in primary human NK cells decreased recruitment of F-actin to the synapse. Live cell imaging experiments showed a linear correlation between NKp46 expression and lytic granules polarization to the immune synapse. Taken together, our data suggest that NKp46 signaling directly regulates the NK lytic immune synapse from early formation to late function.

## Introduction

Natural killer (NK) cells are granular lymphocytes that were initially recognized for their capacity to efficiently eliminate tumor cells without prior sensitization (1, 2). Viral infections can also induce NK cytotoxicity (3). Indeed, NK cells are an important component of the innate immune response against viruses, as evidenced by the abundant infections in patients with primary NK cells deficiencies and mice lacking NK cells (4, 5). In healthy human adults, NK cells compromise 5–15% of peripheral blood lymphocytes (6). The majority of NK cells (~90%) are CD56^dim^ CD16^bright^, and can induce a strong cytolytic response, while 10% are CD56^bright^–CD16^null/dim^ and are capable of rapid cytokine secretion (7). Control of NK cell function is mainly regulated by is recognition of self MHC class I molecules through a set of inhibitory receptors – the killer-cell immunoglobulin-like receptors (KIRs) in humans and the Ly49 receptor family in mice (8–10). NK cells do not express variable antigen specific receptors. Instead, their activation is controlled by a repertoire of germ-line encoded activating receptors that recognize different ligands on the surface of susceptible target cells. Important NK activating receptors include NKG2D (11) and three members of the natural cytotoxicity receptors group (NCR): NKp46 (12), NKp44 (13), and NKp30 (14).

Distinct among the NCRs, NKp46 (NCR1) is evolutionary conserved between mice and humans (15), and NKp46 activity has been studied in several mouse models (16–19). NKp46 is uniquely expressed on all NK cell subsets and has been suggested as a possible target for NK cell ablation and as a pan NK cell marker (19, 20). NKp46 is a 46 kDa type 1 transmembrane glycoprotein characterized by a 30 a.a. intracellular tail, 20 a.a. transmembrane domain, and two extracellular Ig-like domains that are contacted through a 25 a.a short peptide (21). The transmembrane domain contains an Arg residue that can bind the signal adapter proteins FcϵRI and CD3ζ (22). Two NKp46 extracellular (D1–D2) crystal structures were resolved and showed similarity to the extracellular domains of different leukocyte immunoglobulin-like receptors (LIRs) and KIR receptors (23, 24). The membrane-proximal domain (D2) was identified as the ligand-binding domain of NKp46 receptor (25).

To date, few pathogen-associated ligands and cellular co-ligands for NKp46 have been documented. Viral hemagglutinin (HA) of influenza and Sendai viruses (26, 27), and Newcastle disease HA-neuraminidase (28) can bind to NKp46 and induce NK activation. The intermediate filament protein Vimentin, expressed on *Mycobacterium tuberculosis*-infected monocytes, is involved in binding to the NKp46 receptor (29). Target cell-expressed heparan sulfate (HS) can bind with NKp46 and other NK receptors and serves as an accessory molecule for the recognition of cellular ligand(s) by NKp46 (30, 31). On the other hand, NK cell membrane-expressed HS proteoglycans may regulate the function and fate of NKp46 and other HS-binding NK receptors (32). Nevertheless, considering the important role of the NKp46 receptor in tumor cell, it is expected that one or more cancer-associated cellular ligands for NKp46 remains to be discovered (33).

In addition to the role of NKp46 in recognition of cancer cells, several studies suggest the involvement of NKp46 in other pathological conditions. NKp46 was shown to be crucial for development of type 1 diabetes through recognition of an unknown ligand on pancreatic β-cells (34, 35). We demonstrated that NKp46 contributes to clearance of *Streptococcus pneumoniae* by interacting with infected alveolar macrophages (36). NKp46 also mediates the killing of stellate cells in attenuated liver fibrosis (37) and of HCV-infected hepatocytes (38). In a murine model of random mutation (Ncr1^Noé/Noé^), the lack of NKp46 surface expression resulted in hyper-responsiveness of NK cells following MCMV infection (18). We recently demonstrated that targeting of NK cells using an NKp46 antibody can attenuate type 1 diabetes progression in mice (39). NKp46 also regulates graft-versus-host disease (40) and allergic response (41).

Although early studies of NK cells clearly showed that cytolytic activity is contact dependent (2), it was not until two and a half decades later that the intercellular complex between NK and target cells was thoroughly characterized, and the term NK cell immune synapse was coined (42). NK cell immune synapse formation and function is composed of several highly regulated stages (43). However, to date, the role of NKp46 in formation and function of the immune synapse has not been determined.

In our current study, we investigated the role of human NKp46 in NK immune synapse formation and function. We report that following the initiation of an NK-target cell interaction, NKp46 clusters at the cell membrane, specifically at the immune synapse. At the immune synapse, NKp46 mediates cytoskeletal rearrangement and cellular polarization. These results shed light on the specific function of NKp46 in cytotoxicity.

## Materials and Methods

### Cell lines and antibodies

NK92, a human NK lymphoma cell line (CRL-2407) was obtained from ATCC. NK92 cells, expressing recombinant NKp46 or NKp46-IRES-GFP (designated as NK92.p46 or NK92.p46-IRES-GFP, respectively) were kindly provided by Kerry S Campbell (Fox Chase Cancer Center, Philadelphia, PA, USA). NK92 cell lines were grown in MEM Alpha medium (Gibco, Life Technologies), supplemented with heat-inactivated 10% horse serum, 10% FBS (Serum Source International), 0.2 mM myo-inositol (Sigma), 0.1 mM β-mercaptoethanol (Sigma) 0.02 mM folic acid (Fisher Scientific), 200 IU/mL of recombinant human IL-2 (eBioscience), and 50 IU/mL penicillin/streptomycin (Life Technologies) The following target cell lines were used HeLa, human cervical adenocarcinoma (ATCC CCL-2); HepG2, human hepatocellular carcinoma (ATCC HB-8065); 721.221, EBV-transformed human B-cell lymphoma. Cell lines were grown in a 5% CO_2_ humidified 37°C incubator and cultured in RPMI 1640 (Mediatech, Inc.) or DMEM (Gibco, Life Technologies) medium supplemented with 10% FBS and 1% penicillin/streptomycin. Antibodies that were used in this study were anti-human NKp46 PE or Biotin (Biolegend, clone 9E2), anti-human CD3 FITC (BD Pharmingen, clone UCHT1), and anti-human CD56 PE-Cy5 (BD Pharmingen, clone B159).

### Isolation and culture of primary human NK cells

Natural killer cells were isolated from the peripheral blood of healthy donors using the RosetteSep Human NK Cell Enrichment Cocktail (StemCell Technologies). The purity of CD3^−^CD56^+^ NK cells was >95%. Purified NK cells were cultured in CellGro stem cell serum-free growth medium (CellGro) supplemented with 10% heat-inactivated human serum from healthy donors, 1 mM sodium pyruvate, 2 mM l-glutamine, 1× MEM non-essential amino acids, 1% penicillin/streptomycin, 10 mM HEPES (Life Technologies), and 300 IU/mL recombinant human IL-2 and used in experiments within 7 days. Blood donations from healthy volunteers were collected with informed consent, approved by the Stanford University Institutional Review Board.

### Immunocytofluorescence conjugation assay

For experiments with adherent target cells, cells were harvested using 0.05% EDTA cell detachment solution to avoid ligand degradation by trypsin. Target cells were incubated on cell culture-treated eight-well μChamber slides (Ibidi) for 4–6 h at 37°C, 5% CO_2._ Effector cells were added at a 2:1 effector:target ratio and co-incubated for an additional 20 min. Cells were rinsed and fixed using 1.6% v/v para-formaldehyde. Samples were blocked using 3% w/v bovine serum albumin for 30 min and incubated with 10 μg/mL biotinylated monoclonal antibody or isotype control biotinylated antibody in PBS containing 0.05% v/v Triton X-100. Samples were washed thoroughly and incubated with streptavidin Alexa Fluor 647 for 30 min at RT. For F-actin labeling, samples were incubated in PBS solution containing 10 units/mL Rhodamine Phalloidin (Life Technologies), and 0.05% v/v Triton X-100. Nuclei were stained with 2 mg/mL Hoechst 33342 (Sigma) and ProLong Gold antifade (Life Technologies) reagent was added to the samples before mounting. Images were acquired using LSM510 Meta (Zeiss) laser scanning confocal microscope equipped with a 63× (NA 1.4) DIC oil objective.

### Live cell imaging microscopy

Target cells were labeled with DiD cell labeling solution (Life Technologies) according to manufacturer instructions and cultured on poly-l-lysine (Sigma-Aldrich) coated Delta T dishes (Bioptechs Inc.) for 1 h at 37°C in phenol free RPMI supplemented with 10% FBS. NK92.p46.GFP effector cells were loaded with 1 μM of LysoTracker DND-99 (Life Technologies) for 30 min, washed, and re-suspended in phenol free RPMI medium containing 200 IU/mL IL-2. Target cells were placed on a heated Delta T stage adapter with a heated lid, and time-lapse images (6 frames/min) of random fields containing target cells were acquired. After imaging had commenced, effector cells were introduced at a 2:1 effector:target ratio. Images were collected on a Nikon Eclipse Ti fluorescence microscope equipped with a CSU-X1 spinning disk confocal head (Yokogawa), Nikon 100X oil objective (NA 1.4), and an XR/MEGA-10 intensified CCD camera (Stanford Photonics). The system was controlled with Micro-Manager software (44). Nikon Perfect Focus was used to correct drift during time-lapse acquisition.

### Image analysis

All image analysis was done using Fiji software (45). In order to calculate receptor accumulation at the immune synapse, threshold images of the appropriate channel were analyzed for particles larger than 0.09 μm^2^. Total pixel intensity values (i.e., Integrated Density in ImageJ) for each particle were measured. Localization of particles to the immune synapse was determined by overlay of the particle border over the brightfield image of the conjugate. Accumulation of receptor at the immune synapse was than calculated as ratio between Integrated Density values of particles at the immune synapse to the total Integrated Density of the receptor. F-actin accumulation at the immune synapse was calculated as the ratio of immune synapse MFI to total conjugate MFI. A more detailed methodological description can be found in supplemental materials. Lytic granules polarization time was calculated for single effector cell and target cell interactions. NK cells were divided into slices by an X shape centered in the middle of the cell. Lytic granules polarization time was set to the first frame in which 80% of the lytic granule signal (i.e., Integrated Density of the appropriate channel) was concentrated in the slice facing the target cell.

### NKp46 knockdown by siRNA

Silencing of NKp46 expression in primary human NK cells was done using the Amaxa Nucleofector II device. The following siRNA molecules were obtained from Dharmacon: LU020866 ON-Targetplus Human NCR1 set of four sequences (AGACGGGACUCCAGAAAGA, GGAGAAGGCUGAACACACA, GUACAGCGCGGAUACGGGA, and AAUACCAGCUGCACUUUGA), and ON-TARGETplus non-targeting siRNA #1 (UGGUUUACAUGUCGACUAA). 5 × 10^6^ primary NK cells were suspended in Nucleofector solution (Lonza) containing 200 pmol siRNA and transfected using program U-001 according to the manufacturer’s instructions. Transfection was repeated 24 h later. Cell viability and surface expression of NKp46 was verified on a FACScan instrument (BD Bioscience) 72 h post initial transfection. FACS data were analyzed using FlowJo (Treestar, Inc.).

### Statistical analysis

Graphics and statistical analysis were performed using GraphPad/Prism6 software. Statistical significance was tested using the two-tailed Mann–Whitney test. Correlation was tested using Spearman’s rank correlation coefficient. *p* < 0.05 was considered statistically significant.

## Results

### NKp46 forms microclusters at the cytolytic NK cell immune synapse

**Figure 1:**
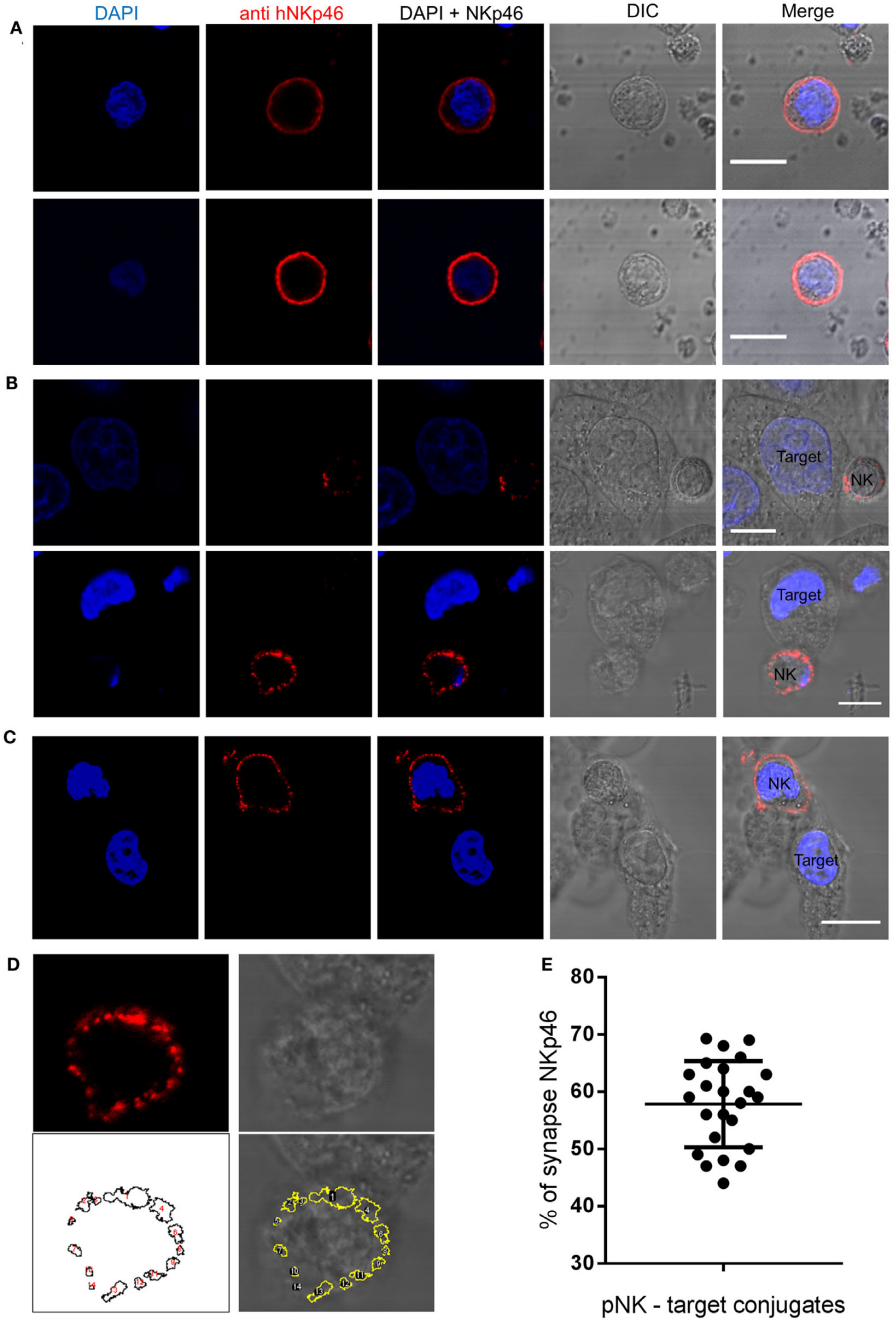
**NKp46 spatial distribution at the immune synapse**. Representative images of fixed human primary NK cells stained with DAPI for nuclear labeling (blue), anti-NKp46-biotin followed by streptavidin Alexa Fluor 647 (red), and DIC images. **(A)** Non-interacting primary NK cells. **(B,C)** NK cells co-cultured with target HeLa cells; with **(B)** or without **(C)** accumulation at the immune synapse. **(D)** NKp46 distribution analysis using automated particles detection. Upper panels show the enhanced magnified image from **(B)** of NKp46 staining (left) and DIC (right). The lower left panel shows detected objects in a masked image. Overlay is shown in lower right panel. Object 1 is the synapse area. **(E)** Summary of relative accumulation of NKp46 at the immune synapse from 24 NK-target interactions. Scale bars = 10 μm.

Organization of NK receptors in activating clusters is important for signaling (46). To examine the spatial distribution of endogenous NKp46 in activated primary human NK cells, we used a monoclonal antibody to NKp46 to examine NK cell interactions with HeLa target cells, The NKp46 receptor distributes homogenously on the cell membrane surface of NK cells that do not form immune synapses with target cells (Figure 1A). In contrast, confocal images of NK cells that bind and interact with target cells clearly demonstrate that the NKp46 receptor segregates in large clusters at the immune synapse (Figure 1B). In a few observed conjugations (3 out of 27), the NKp46 receptor segregated at the cell membrane but did not cluster at the immune synapse (Figure 1C). In order to quantify the portion of the receptor molecules that are accumulating at the immunological synapse, we used automated particle analysis. In one representative image (enhanced magnification of Figure 1B, lower panel), 14 objects were detected (white numbers), with objects one and four in the area of the immune synapse according to NK-target cell contact area in brightfield image (Figure 1D). Measuring the distribution of labeled NKp46 in 24 NK-target cell conjugations indicated that 58 ± 8% (mean ± SD) of the membrane-associated NKp46 receptor molecules are accumulating at the immune synapse (Figure 1E). These results clearly indicate that following NK cell conjugation, NKp46 segregates into distinct domains at the cell membrane. The enrichment of clusters at the immune synapse suggests that the receptor confinement is important for activation.

### NKp46 over-expression increases F-actin recruitment to the immune synapse

**Figure 2:**
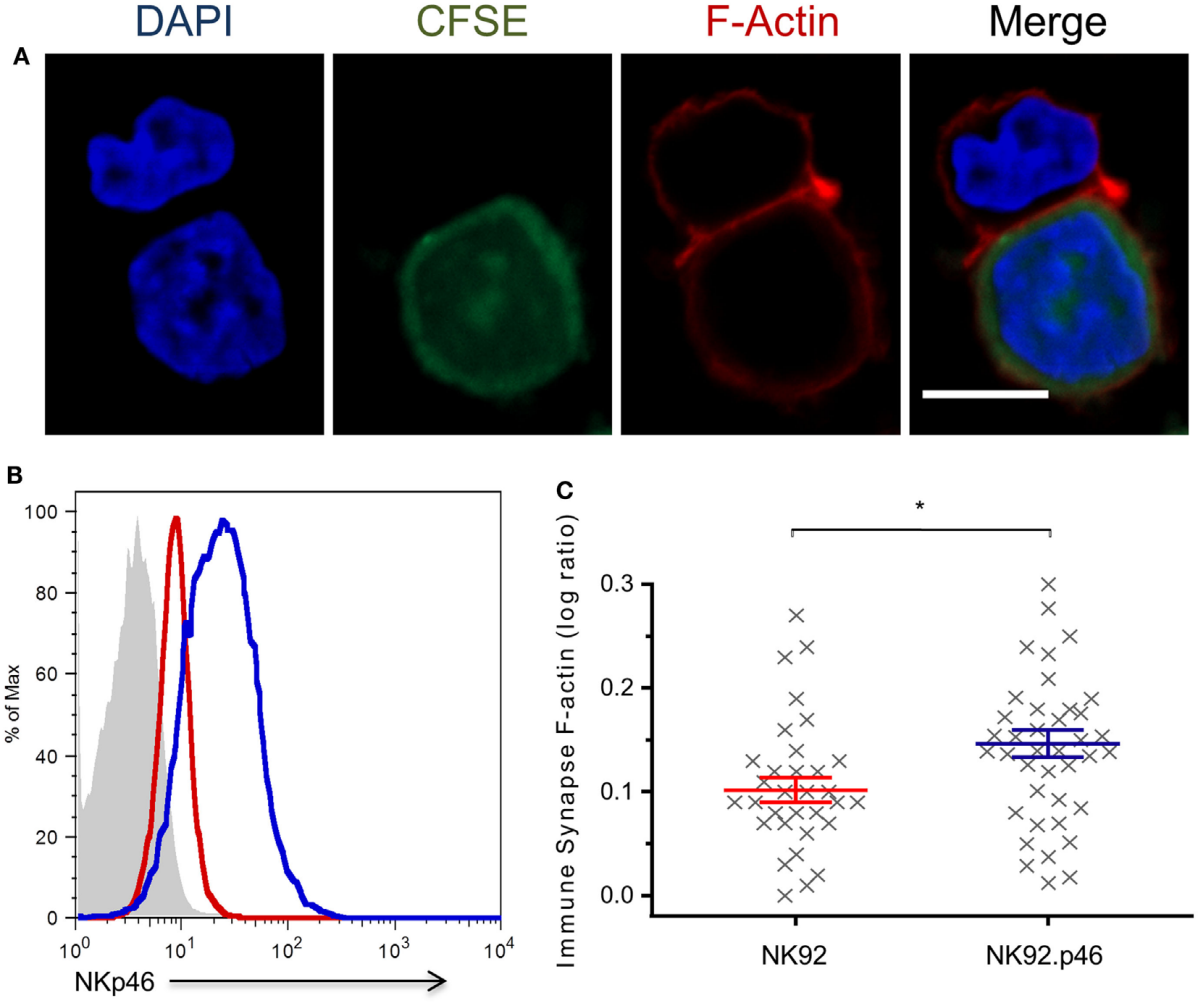
**NKp46 over-expression increases F-actin rearrangement at the immune synapse**. **(A)** Representative micrograph of NK92 cell co-incubated with CFSE-labeled (green) target cell, fixed and permeabilized, and stained for F-actin using Rhodamine Phalloidin (red) and DAPI (blue) for nucleus labeling. Scale bar = 10 μm. **(B)** Flow cytometry analysis of NKp46 expression NK92 (dashed line), NK92.p46 (solid line) cells, and isotype antibody staining (gray-filled graph). **(C)** Calculated ratio of synapse specific F-actin (Figure S2 in Supplementary Material) is presented for multiple effector-target conjugations from three independent experiments (*x*, *n* = 32) as log values and is representative of three repeated experiments. Graph bars show mean ± SEM. *p < 0.05, Student’s *t*-test.

Cytoskeletal rearrangement that leads to formation of a dense F-actin mesh at the immune synapse is a central step in NK cell-mediated cytotoxicity (42). Over-expression of NKp46 increases NK cytotoxicity against HeLa cells (Figure S1 in Supplementary Material). To test the specific effect of NKp46 signaling on F-actin recruitment to the immune synapse, we developed a quantitative conjugation assay that allowed us to measure F-actin accumulation at the immune synapse (Figure 2A; Figure S2 in Supplementary Material). Staining of NK-target conjugations with Phalloidin show that NKp46 over-expression (NK92.p46; blue) in an NK cell line increased the amount of F-actin at the immune synapse by 30% as compared to staining of low NKp46 expressing cells (NK92; red), suggesting that cytoskeletal rearrangement is influenced by NKp46 signaling (Figures 2B,C). Importantly, there were no significant differences in the expression of other NK cell-activating receptors between NK92 and NK92.p46 cells (Figure S3 in Supplementary Material).

### Knock-down of endogenous NKp46 impairs cytoskeletal rearrangement

**Figure 3:**
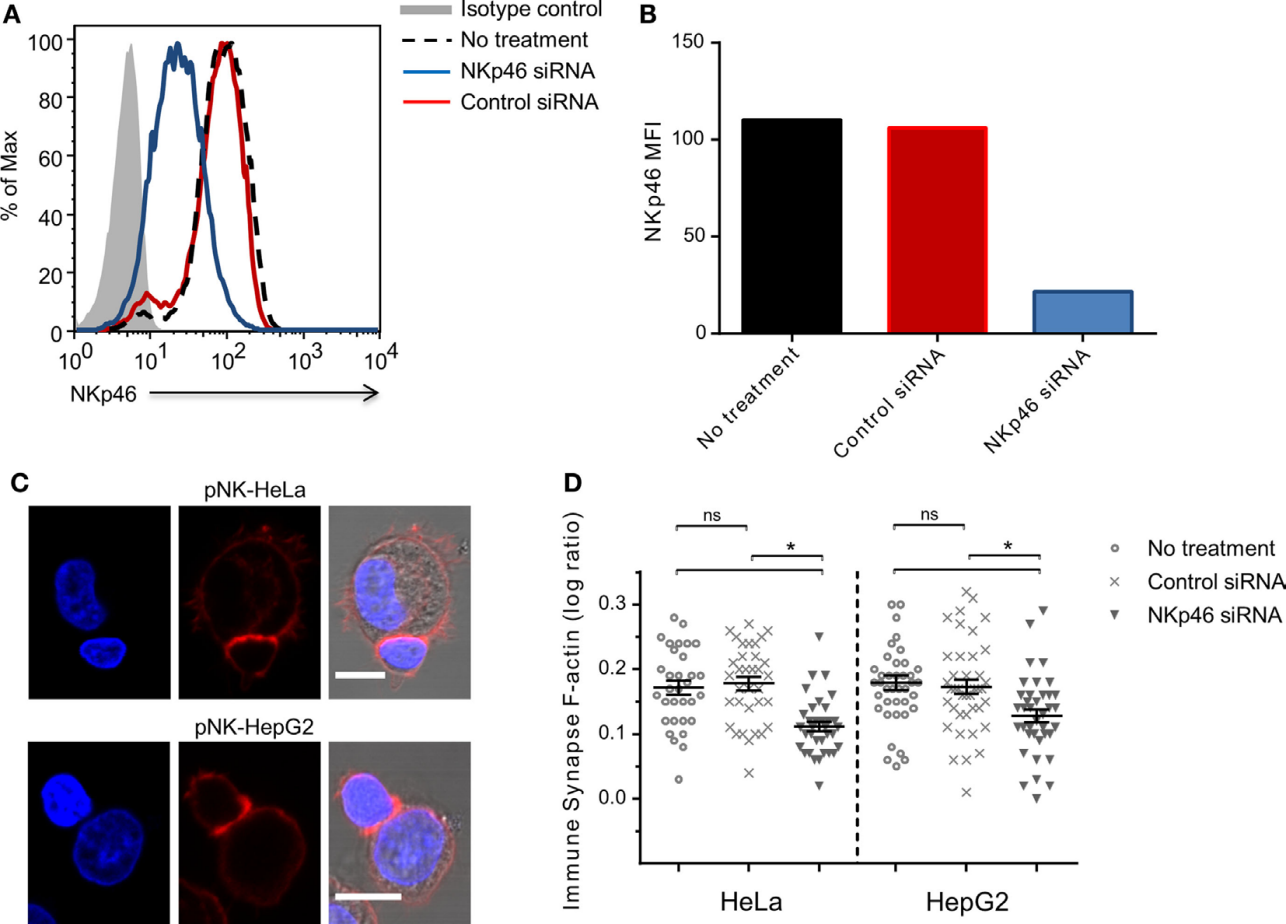
**NKp46 knockdown in primary NK cells modifies F-actin accumulation**.**(A)** NKp46 surface expression analysis 72 h post NKp46 siRNA (blue solid line), negative control siRNA (red solid line), untreated primary NK cells (black dashed line), and isotype control staining (gray-filled graph). Scale bar = 10 μm **(B)** Summarized MFI for surface NKp46 in NKp46 siRNA (blue), negative control siRNA (red), untreated primary cells (black) treated cells. **(C)** Representative micrographs of NKp46 siRNA-treated primary NK cells that were incubated with HeLa (upper panel) or HepG2 (lower panel) target cells, fixed and permeabilized, and stained for F-actin (red) and DAPI (blue). **(D)** Results summarized for different effector–target interactions synapse (*n* > 30) log values F-actin ratios of representative experiment of two repeated experiments Graph bars show mean ± SEM. *p < 0.05, Student’s *t*-test.

To validate the role of endogenous NKp46 in rearrangement of the cytoskeleton, we knocked down NKp46 in primary human peripheral blood NK cells using siRNA. Analysis of cell surface NKp46 following electroporation showed that NKp46 siRNA (blue line) almost completely abolished receptor levels, while control siRNA (red line) has no effect (Figures 3A,B). Importantly, there was no observed difference in cell growth or morphology between the control siRNA group and NKp46 siRNA group (data not shown). Analysis of interactions between NK cells and HeLa or HepG2 cells showed that knockdown of NKp46 resulted in the recruitment of 40 and 45%, respectively less F-actin to the immune synapse, whereas NK cells that received control siRNA recruited the same amount of F-actin to the immune synapse as untreated NK cells (Figures 3C,D). These results further support the observation that NKp46 contributes to cytoskeletal rearrangement at the immune synapse.

### NKp46 expression linearly correlates with reduced lytic granules polarization time

**Figure 4:**
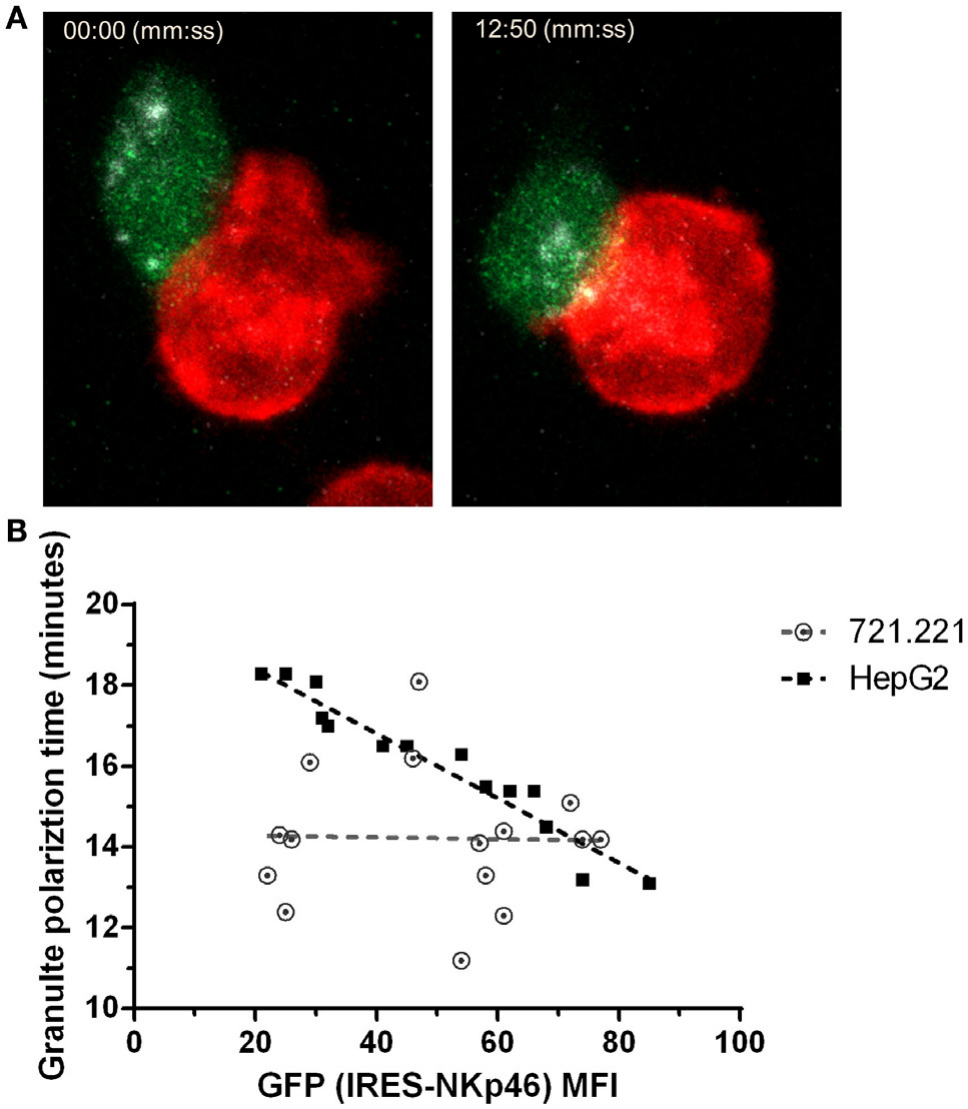
**NKp46 expression correlates with reduced lytic granule polarization time**. **(A)** Representative snapshots from live cell time-lapse imaging (Video S1 in Supplementary Material). NK92 cells expressing NKp46-IRES-GFP (green) were pre-loaded with LysoTracker DND-99 (gray) and co-incubated with DiD-labeled (red) 721.221 or HepG2 cells. Left panel shows time of initial effector–target interaction and right panel shows time of lytic granules polarization. **(B)** Summarized results for multiple individual effector-target show lytic granules polarization time per NK cell GFP MFI. Dashed lines show trends adapted by linear correlation test. Scale bar = 10 μm.

Distinct from cytotoxic T cells, NK cell induced cytolytic killing is largely dependent on microtubule-organizing center (MTOC) followed by lytic granule polarization to the immune synapse (47). To explore the effect of NKp46 surface expression on lytic granules polarization at the single cell level, we used NK92 cells expressing an NKp46-IRES-GFP construct in which GFP is a reporter for NKp46 expression level. We validated that GFP intensity correlates with surface expression of NKp46 (Figure S4 in Supplementary Material). Using NK cells pre-loaded with LysoTracker, we measured lytic granule polarization to the immune synapse with respect to time in NK – target cell conjugates using time-lapse live cell imaging (Figure 4A and Video S1 in Supplementary Material). Data collected from multiple interactions (*n* > 15) between NK92.p46-IRES-GFP cells and 721.221 target cells, which express low levels of NKp46 ligand (Figure S5C in Supplementary Material), showed no correlation between NKp46 receptor expression and time to polarization of the MTOC (*R*^2^ = 0.05, slope = −0.002). In contrast, when we analyzed interactions with HepG2 cells, which express high levels of NKp46 ligand (Figure S5A in Supplementary Material), we found that increased NKp46 receptor expression correlated with a linear reduction in the granule polarization time (*R*^2^ = 0.93, slope = −0.08) (Figure 4B). These results show that NKp46 plays a crucial role in the rapid recruitment of lytic granules to the NK-target cell interface.

## Discussion

In this study, we manipulated the expression of NKp46 to test its effect of NK cell immune synapse formation. We show by over-expression of NKp46 in an NK cell line and knock-down in primary NK cells that NKp46 is involved in cytoskeletal rearrangements that lead to formation of a thick F-actin mesh at the NK immune synapse. Accumulation of F-actin at the peripheral supra molecular activating cluster (pSMAC) is an early stage in the formation of functional lytic NK cell immune synapses (48, 49). This accumulation is important for firm NK-target cell adhesion, actin dependent signaling, and perforin polarization (43). The fact that NKp46 contributes to F-actin accumulation, implies that it engages with its unknown cellular ligand at an early time point of NK–target cell interaction. It also suggests that NKp46 is involved in the initial recognition of target cells by NK cells. Improved identification of the NKp46 ligand(s) will allow us to test these hypotheses.

Intriguingly, an examination of the NK immune synapse by super-resolution microscopy showed that either anti-NKp46 mAb or Influenza HA, together with LFA-1 ligation, can induce spreading of NK cells and generate openings of the actin mesh in the later stages of immune synapse formation (50). This opening in the actin mesh is thought to be essential for lytic granules to reach the cell membrane at the point of interaction with the target cell (50, 51). To further define this pathway, experiments are necessary to directly compare the effect of NKp46 triggering on cytoskeletal rearrangement when bound to its cellular ligand, or to HA. Wiskott–Aldrich syndrome protein (WASp) is expressed in hematopoietic cells and facilitates the reorganization of the actin cytoskeleton (52). Comparison of NK cells from healthy donors and patients with WAS demonstrated that NKp46-dependent signaling and WASp activity are important for the integration of signals leading to nuclear translocation of NFAT2 and NF-κB (RelA) during cell–cell contact (53). This connection between NKp46 and WASp activity may explain the regulation of actin polymerization by NKp46.

Following NK cell activation, there is a rapid convergence of lytic granules around the MTOC (54) and polarization of the MTOC to the immune synapse, which is crucial for NK cell cytotoxicity (55, 56). Our data show that polarization of lytic granules is partially dependent on NKp46 activation and directly correlates with the level of expression of NKp46 when the target cells expresses ligand(s) of NKP46. Interestingly, we did not observe an obvious threshold of activation for this NKp46-mediated effect. Additionally, our data show a similar, gradual polarization of the MTOC to the immune synapse, as previously described (54). The shorter average time for polarization in our experiments (17.5 ± 1.7 min) compared to previous work (36.19 ± 2.4 min) could be explained by methodological differences (54).

Several inhibitory and activating NK cell receptors re-organize in micro- and nano-scale clusters during NK cell immune synapse development. Very early studies of the NK cell immune synapse demonstrated that inhibitory receptor (i.e., KIRs) clusters dominate the inhibitory synapse (42, 57, 58). Phosphorylation of these receptors occurs mainly in these clusters (59). The activating receptors CD2 and CD11a cluster at the mature lytic immune synapse (49), and NKG2D receptors cluster at ganglioside GM1-rich micro-domains (60). Our data show that following interaction with a target cell, endogenous NKp46 on the surface of primary NK cells clusters at high density at the immune synapse. Interestingly, our data show that small clusters of NKp46 are also present in parts of the NK cell membrane away from the intercellular interface. However, NKp46 clusters are not on the cell membrane of non-interacting NK cells. NK cells from transgenic MHC class I-deficient mice (K^b^D^b^KO mice) and NCR1-eGFP mice (NKLT^+/−^) similarly showed that NKp46 confinement in nanodomains of the cell membrane of non-interacting cells is largely controlled by actin cytoskeleton dynamics (61). However, the mechanism that controls NKp46 segregation at the immune synapse remains unknown. Previous studies demonstrated that adaptor proteins and signaling molecules segregate into activating clusters, thus providing physical proximity that enables signal amplification and integration at the lytic immune synapse (57, 62, 63). Knock-down of FcϵRI, but not CD3ζ, reduces surface expression of NKp46 in non-interacting primary human NK cells. Moreover, a complex consisting of NKp46 and FcϵRI is required for 2B4-mediated cytotoxicity (64). Taken together, these results suggest that the NKp46 activating complex includes the adapter protein FcϵRI, and possibly the 2B4 receptor. It is conceivable that this activating complex is also involved in NKp46 clustering at the immune synapse.

Previously, we described the homo-dimerization binding site (aa 136–155) at the NKp46 membrane-proximal ectodomain. We also found that dimerization can only occur in the presence of the NKp46-derived HS binding peptide, and hypothesized that this interaction is involved in switching the receptor from an inactivate to activate state (65). Our current data demonstrating the involvement of NKp46 in F-actin rearrangement suggests that the receptor switching occurs in the early stages of the interaction with the target cell. The potential interaction of NKp46 with cell adhesion molecules CD2 (LFA-2) could provide such an activation mechanism (66). Inside-out signaling by integrins at the immune synapse can also induce an early rapid switching of receptors (67).

In this study, we used both over expression and knock-down strategies to demonstrate the importance of NKp46 in NK cell polarization during interactions with target cells. Our previous studies, using siRNA knock-down of NKp46, suggested that killing of immature DCs is independent of NKp46 activity in rat NK cells (68). However, studies with blocking antibodies to NKp46 (69, 70) NKp46 KO mice indicate that NKp46 can be involved in immature DC killing (40, 68). We, and others, have also found that NKp46 mediates secretion of IFN-γ in interactions with virally infected (71, 72) or mature DCs (68). When interacting with other cells of the immune system, mainly DCs, NK cells form regulatory immune synapses (73–75). In this type of synapse, activating receptors aggregate, but there is no MTOC polarization (76). Further studies of NKp46 function in the regulatory immune synapse may help elucidate its role in NK cell-DC cross-talk.

Our findings further support the notion that different NK cell-activating receptors can prompt parallel effector functions. Despite intensive research in the field, the biological basis for the vast redundancy in NK cell-activating receptors is still unclear. In some cases, synergy between activating receptors was shown to be vital for potent action of the cell (51, 66). For NKp46, the co-ligation of CD2 is required to induce degranulation in resting NK cells (66). Furthermore, study of individual receptors revealed that many NK activating receptors can provide inside-out signaling that switch LFA-1 to an open state (77). In some studies, LFA-1 has been shown to be solely responsible for cytoskeleton rearrangement (62). Thus, it remains possible that in our system, cytoskeletal rearrangement is indirect through LFA-1 switching, rather than directly dependent on NKp46 signaling. This type of synergy and hierarchy among NK cell receptors may provide the necessary control over activation of NK cells, which lack the specificity of cytotoxic T cells. Another explanation for the abundance of seemingly redundant NK cell-activating receptors comes from a recent study that revealed an overwhelming diversity in the NK cell populations in an individual donor (78). Although the functional meaning of these ultra-specific NK cell populations remains to be elucidated, such diversity may achieve specific recognition of pathogen infection or stress induced changes in cells.

Dissecting the function of NKp46 in NK cell polarization clarifies the downstream cellular functions that are regulated by its activity. Our studies of NKp46 clarify that NCR receptors play an important role in NK cell killing and support efforts to target these molecules therapeutically.

## Author Contributions

UH designed the study, performed the experiments, analyzed the data, and wrote the manuscript. SK supervised the project, analyzed the data, wrote and edited the manuscript. TT, OM, MB, and AP provided critical notes to the manuscript. TT and MB advised on microscopy experimental procedures.

## Conflict of Interest Statement

The authors declare that the research was conducted in the absence of any commercial or financial relationships that could be construed as a potential conflict of interest.

## Supplementary Material

The Supplementary Material for this article can be found online at http://journal.frontiersin.org/article/10.3389/fimmu.2015.00495

Figure S1 **NK92 and NK92.46 cytotoxicity**. Lysis of HeLa cells by NK92 and NK92.46 was performed using 7AAD incorporation and flow cytometry as previously described (79). Briefly, NK cells were incubated with DiO-labeled HeLa cells for 5 h at the indicated ratio, 7AAD was added to samples and analysis was performed using a FACSCanto II (BD Biosciences). Specific lysis was calculated as compared target cells that were not co-incubated with NK cells.

Figure S2 **Relative quantification of immune synapse specific F-actin accumulation**. NK cells were co-incubated on confocal chamber slides with CFSE-labeled target cells (green), fix and permeabilized, and stained with Rhodamine Phalloidin. In representative images of NK-target interactions shown in **(A)**, pseudo color (black to white) was applied to indicate Rhodamine Phalloidin fluorescence intensity. Upper panel shows conjugation with relatively high levels of immune synapse F-actin and lower panel shows low level of F-actin at the immune synapse. Images with saturated pixels were not use for analysis. **(B,C)** For image analysis, background fluorescence noise was eliminated using ImageJ mean threshold algorithm (indicated by blue background). Pixels below threshold are excluded from calculation. In order to eliminate the florescent signal originated from target cell F-actin and variation in staining intensity, gated synapse F-actin MFI **(B)** was divided by total conjugation MFI **(C)**. For statistical significance calculation, log values of ratios were used as following: ${\text{log}}_{10}\left(\frac{\text{Synapse MFI}}{\text{Conjugation MFI}}\right).$

Figure S3 **Expression of surface-activating receptors on NK92 cell lines**. NK92 cells were incubated with either anti-NKp30 PE-conjugated, anti-NKp44 PE-conjugated, or anti-NKG2D APC-conjugated monoclonal antibodies (solid black line) with specific matching isotype controls (gray filled). Acquisition was done using FACSCanto II (BD Biosciences).

Figure S4 **Microscopy measurement of NKp46 expression level in single cells using GFP-IRES expression**. **(A)** NK92 cells, stably expressing the NKp46-IRES-GFP vector were stained by isotype control (left) NKp46 PE-conjugated monoclonal antibody (right) and analyzed by flow cytometry. **(B)** Fluorescence micrograph of NK92.p46-IRES-GFP (green) cells overlaid with DIC image shows variation in GFP expression. Scale bar = 10 μm.

Figure S5 **NKp46 ligand expression analysis in target cell lines**. **(A)** HepG2 **(B)** HeLa **(C)** 721.221 target cell lines were stained using either NKp46-human Ig fusion protein (solid black line) or control human Ig protein (gray-filled graph) as previously described (65). Cells were stained with secondary anti human Ig APC-conjugated antibody and analyzed by flow cytometry.

Video S1 **Lytic granules tracking in live cell imaging microscopy**. Target cells were pre-labeled with DiD membrane dye (red) and co-incubated with NK92.p46-IRES-GFP (green) pre-loaded with LysoTracker DND99 (gray) on a δT dish at 37°C. Time lapse images of random fields were recorded over 30–90 min at frame per 10 s rate. Granules polarization time was determined by minimal distance to the immune synapse as previously described (Mentlik MBOC 2010). Representative composite video shows time from initial interaction at 7 frames/s rate.

## Funding

Supported by NIH AI10423 (SK), a Stanford Institute of Immunity, Transplantation and Infection (ITI) seed grant (UH and SK), and by the TTS Basic Science Research Exchange Fellowship for Trainees Award (UH).

## Abbreviations

ADCC, antibody-dependent cellular cytotoxicity; HA, hemagglutinin; HS, heparan sulfate; KIRs, killer-cell immunoglobulin-like receptors; MTOC, microtubule-organizing center; NCR, natural cytotoxicity receptor; pSMAC, peripheral supra molecular activating cluster; WASp, Wiskott–Aldrich syndrome protein.
