# Supplementary figures and images for: NKp46 Clusters at the Immune Synapse and Regulates NK Cell Polarization
Source: Front Immunol. 2015 Sep 25;6:495. doi: 10.3389/fimmu.2015.00495 (PMC4585260; doi:10.3389/fimmu.2015.00495)

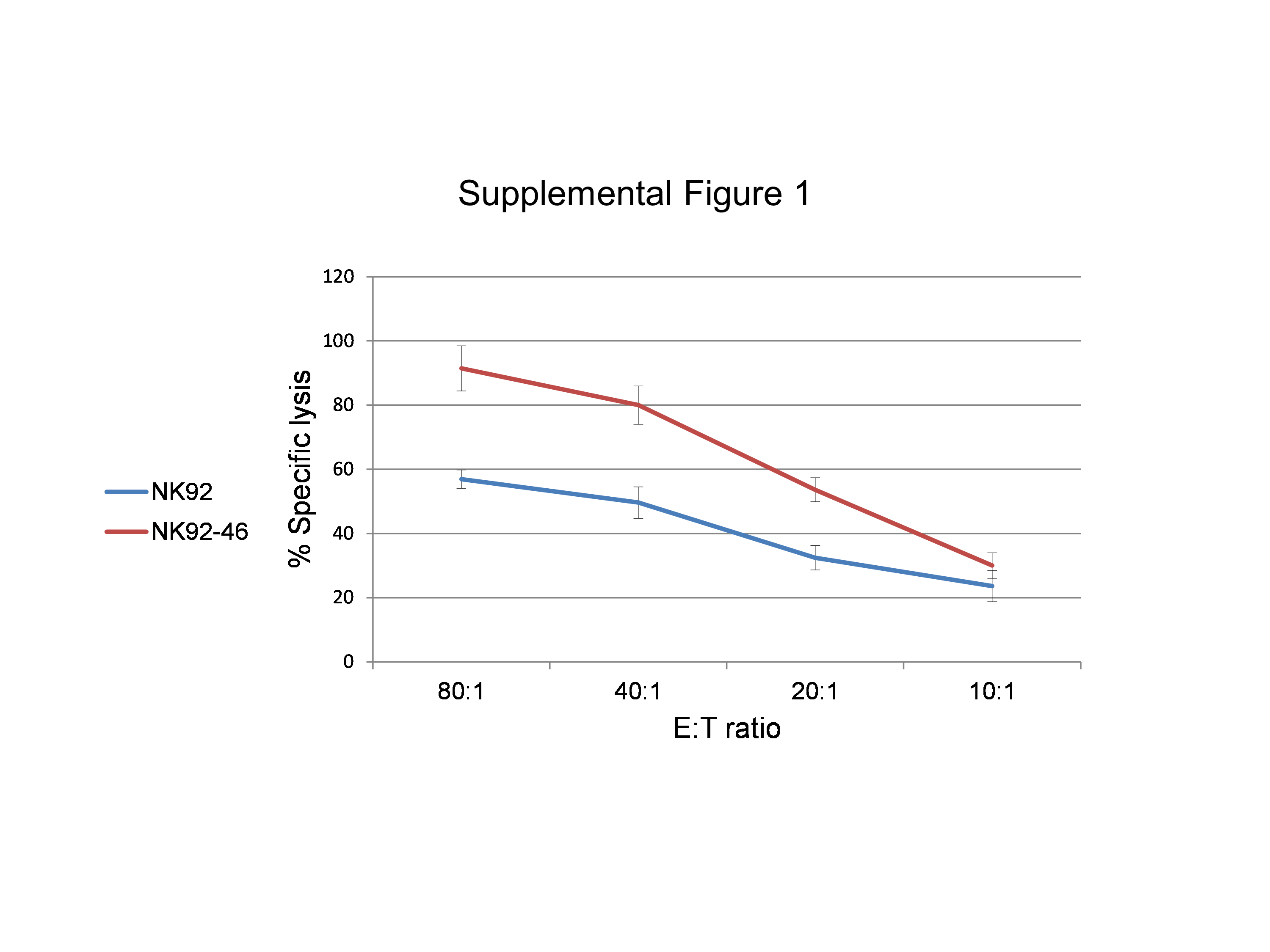

Supplement: Figure S1 — NK92 and NK92.46 cytotoxicity. Lysis of HeLa cells by NK92 and NK92.46 was performed using 7AAD incorporation and flow cytometry as previously described (79). Briefly, NK cells were incubated with DiO-labeled HeLa cells for 5 h at the indicated ratio, 7AAD was added to samples and analysis was performed using a FACSCanto II (BD Biosciences). Specific lysis was calculated as compared target cells that were not co-incubated with NK cells. [file Image_1.TIF]

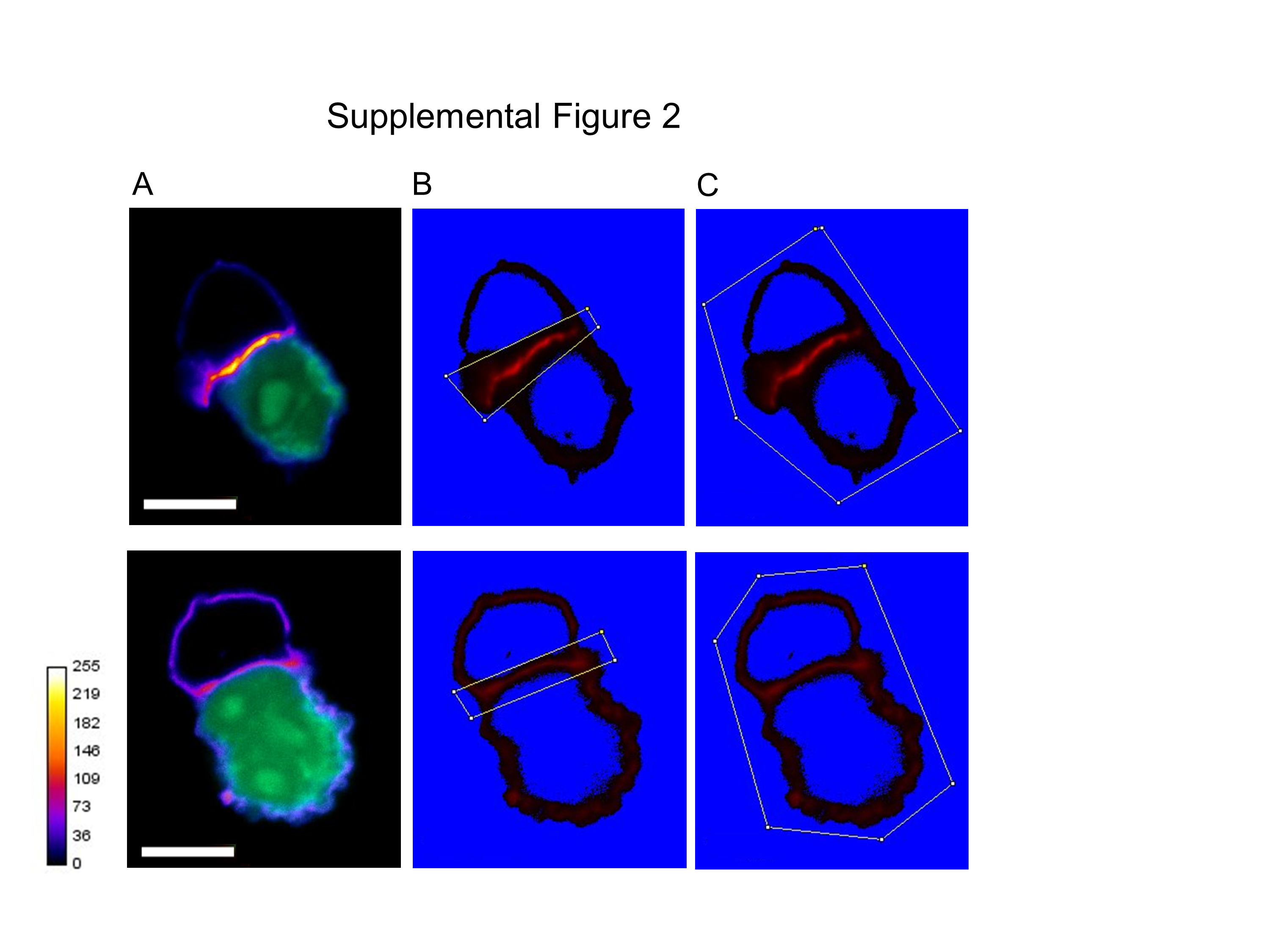

Supplement: Figure S2 — Relative quantification of immune synapse specific F-actin accumulation. NK cells were co-incubated on confocal chamber slides with CFSE-labeled target cells (green), fix and permeabilized, and stained with Rhodamine Phalloidin. In representative images of NK-target interactions shown in (A), pseudo color (black to white) was applied to indicate Rhodamine Phalloidin fluorescence intensity. Upper panel shows conjugation with relatively high levels of immune synapse F-actin and lower panel shows low level of F-actin at the immune synapse. Images with saturated pixels were not use for analysis. (B,C) For image analysis, background fluorescence noise was eliminated using ImageJ mean threshold algorithm (indicated by blue background). Pixels below threshold are excluded from calculation. In order to eliminate the florescent signal originated from target cell F-actin and variation in staining intensity, gated synapse F-actin MFI (B) was divided by total conjugation MFI (C). For statistical significance calculation, log values of ratios were used as following: log10(Synapse MFIConjugation MFI). [file Image_2.TIF]

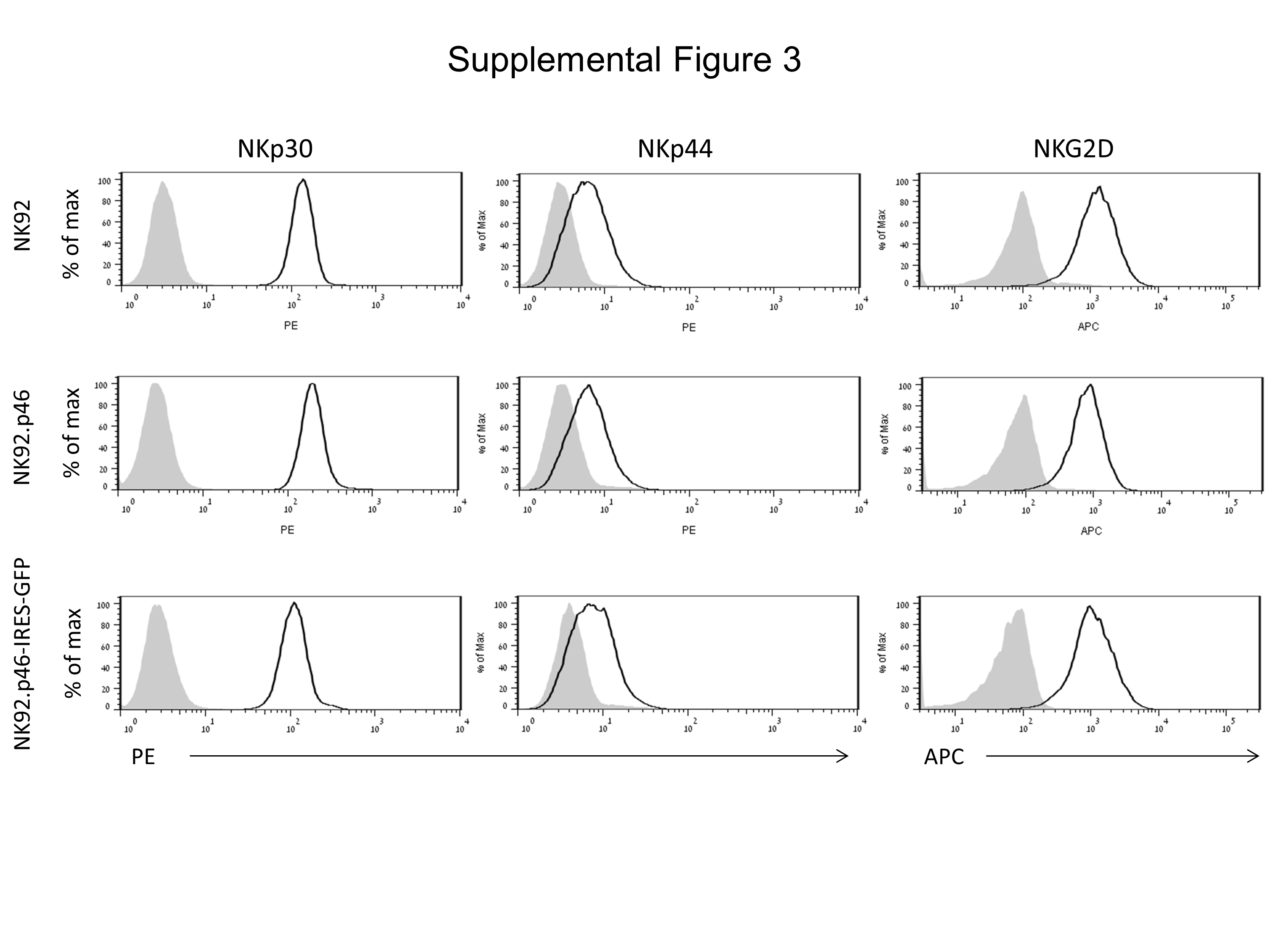

Supplement: Figure S3 — Expression of surface-activating receptors on NK92 cell lines. NK92 cells were incubated with either anti-NKp30 PE-conjugated, anti-NKp44 PE-conjugated, or anti-NKG2D APC-conjugated monoclonal antibodies (solid black line) with specific matching isotype controls (gray filled). Acquisition was done using FACSCanto II (BD Biosciences). [file Image_3.TIF]

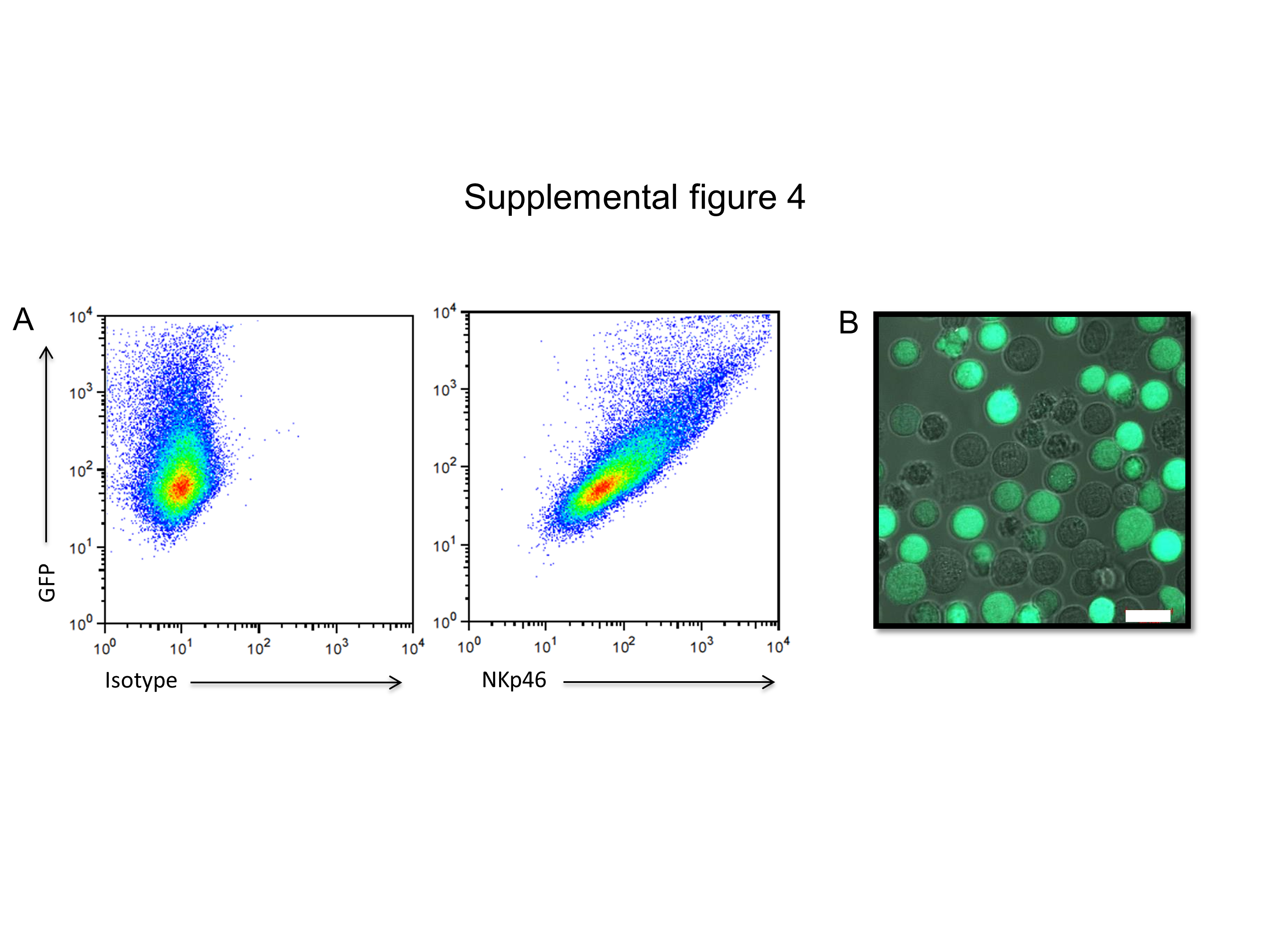

Supplement: Figure S4 — Microscopy measurement of NKp46 expression level in single cells using GFP-IRES expression. (A) NK92 cells, stably expressing the NKp46-IRES-GFP vector were stained by isotype control (left) NKp46 PE-conjugated monoclonal antibody (right) and analyzed by flow cytometry. (B) Fluorescence micrograph of NK92.p46-IRES-GFP (green) cells overlaid with DIC image shows variation in GFP expression. Scale bar = 10 μm. [file Image_4.TIF]

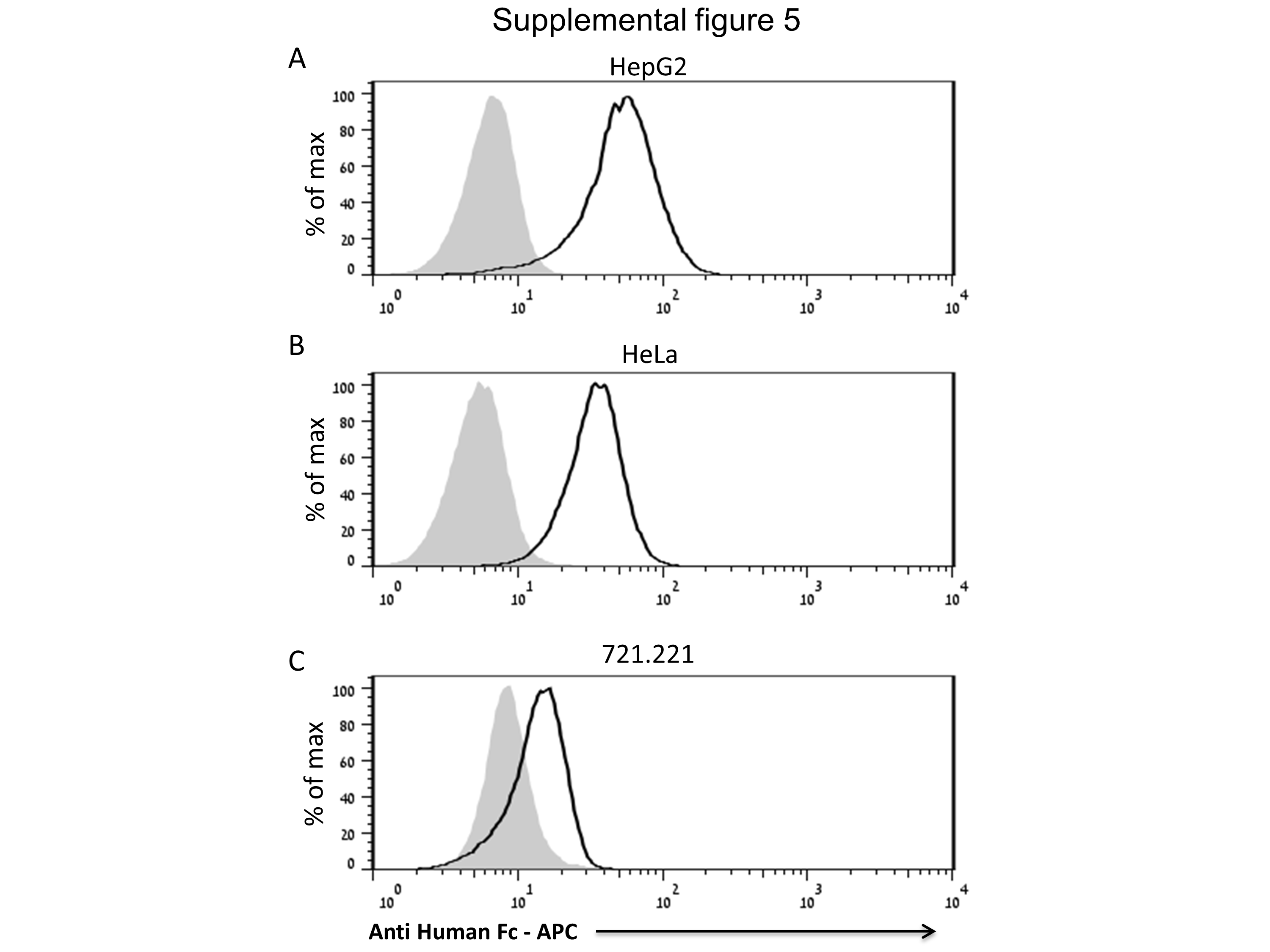

Supplement: Figure S5 — NKp46 ligand expression analysis in target cell lines. (A) HepG2 (B) HeLa (C) 721.221 target cell lines were stained using either NKp46-human Ig fusion protein (solid black line) or control human Ig protein (gray-filled graph) as previously described (65). Cells were stained with secondary anti human Ig APC-conjugated antibody and analyzed by flow cytometry. [file Image_5.TIF]
